# Zerumbone Exhibit Protective Effect against Zearalenone Induced Toxicity via Ameliorating Inflammation and Oxidative Stress Induced Apoptosis

**DOI:** 10.3390/antiox10101593

**Published:** 2021-10-12

**Authors:** Hamad Mohammed AbuZahra, Peramaiyan Rajendran, Mohammad Bani Ismail

**Affiliations:** Department of Biological Sciences, College of Science, King Faisal University, Hofuf 31982, Saudi Arabia; mbaniismail@kfu.edu.sa

**Keywords:** zearalenone, zerumbone, inflammation, oxidative stress, antioxidants

## Abstract

Zearalenone are widely occurring food contaminants that cause hepatotoxicity. This research work aimed to investigate how zerumbone, a plant-derived dietary compound, can fight ZEA-induced hepatotoxicity. ZER is found to increase the cells’ toxin resistance. This study was performed on mice challenged with ZEA. The administration of ZER decreased the level of alkaline phosphatase and alanine aminotransferase (ALT). Simultaneously, ZER attenuated the inflammatory response via significantly reducing the levels of pro-inflammatory factors, including interleukin-1β (IL-1β), IL-6, and tumor necrosis factor-α (TNF-α) in serum. Pretreatment with ZER reduced the hepatic malondialdehyde (MDA) concentration, as well as the depletion of hepatic superoxide dismutase (SOD), hepatic glutathione (GSH), and hepatic catalase (CAT). Moreover, it significantly ameliorated ZEA-induced liver damage and histological hepatocyte changes. ZER also relieved ZEA-induced apoptosis by regulating the PI3K/AKT pathway and Nrf2 and HO-1 expression. Furthermore, ZER increasingly activated Bcl2 and suppressed apoptosis marker proteins. Our findings suggest that ZER exhibits the ability to prevent ZEA-induced liver injury and present the underlying molecular basis for potential applications of ZER to cure liver injuries.

## 1. Introduction

Certain fungi produce toxic secondary metabolites called mycotoxins, characterized by a low molecular weight. Under favorable conditions, these toxins are produced in large quantities and can cause diseases in both human beings and animals through food contamination. Zearalenone [1] is one such mycotoxin produced mainly by the fungal species *Fusarium graminearum* and *Fusarium culmorum* [2]. ZEA is hepatotoxic and induces adverse liver lesions through induction of micronuclei and chromosome aberrations, DNA fragmentation, cell cycle arrest, etc., [3,4]. Research has found that ZEA generates oxidative stress, which is the prime reason for its hepatotoxicity. Notably, a host of liver disorders are caused by reactive oxygen species (ROS). Although the mechanism of ZEA-induced liver injury is complex, it has been proved that inflammatory response is a critical step in the process. Fan et al. [5] found that ZEA activates the secretion of TNF-α, IL-1β, IL-6, and other pro-inflammatory cytokines in IPEC-J2 and macrophages. Therefore, safe and effective natural anti-inflammatory agents are needed for preventing or alleviating ZEA-induced hepatic injury.

**Figure 1:**
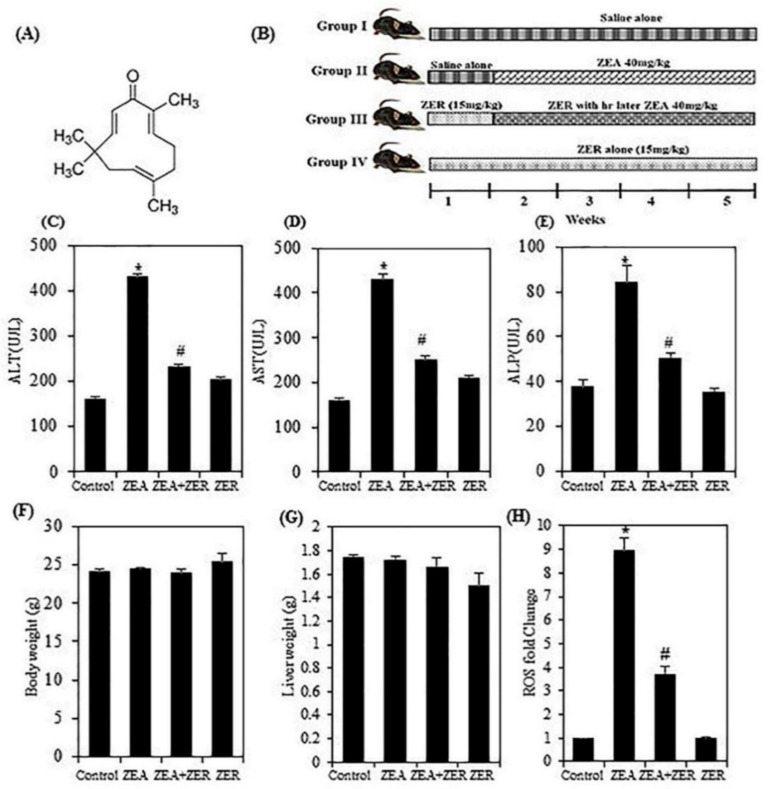
ZEA stimulated ROS levels and ALT, AST, and ALP activation, which were suppressed in ZER-pretreated albino mice. (**A**) Chemical structure of zerumbone. (**B**) Experimental timeline schematic for animal experiments. (I: control; II: ZEA alone; III: ZER with ZEA; IV: ZER alone). (**C**) Effect of ZER on serum alanine transaminase (ALT) activation. (**D**) Effect of ZER on serum aspartate aminotransferase (AST) activation. (**E**) Effect of ZER on serum alkaline phosphatase activity activation. (**F**,**G**) Body and liver weight of control and experimental animals. (**H**) ROS generation in liver tissue of control and experimental animals. H2DCFDA fluorescence employing flow cytometry measured the intracellular ROS levels. Data are expressed as the mean values ± SEM of independent experiments (*n* = 6). * *p* < 0.05 represents significant variations in the ZEA alone group compared with the control group. # *p* < 0.05 represents significant variations compared the ZEA alone and ZER with ZEA treatment groups.

The detrimental effects of mycotoxins such as ZEA can be alleviated using certain phenolic compounds and herbal extracts [6,7]. One such compound is zerumbone [8] (Figure 1A). It is extracted from wild ginger called *Zingiber zerumbet*, a subtropical Zingiberaceae family commonly known as Southeast Asian ginger. ZER is a monocyclic sesquiterpene with a cross-conjugated dienone system. Zerumbone, which comprises approximately 31.7% of *Z. zerumbet*, is extracted using gas chromatography. *Z. zerumbet* has always been an important component of folk medicine; however, zerumbone has gained popularity in modern medicine for its pharmacological activity against certain pathological conditions [9]. The LD 50 value of ZER is 1.84 g/kg [10] and for human (LD50 = 42.4 µg/adult) [11]. The added advantage of ZER is that it does not affect normal cells (HaCaT) [9] but can harm cancer cells (0–786) [12]. Moreover, ZER exhibits antinociceptive, anti-inflammatory, antitumor, antiproliferative, and antiplatelet aggregation properties [12,13,14,15,16]. However, its effect on mycotoxins has not been studied in detail. Thus, this study aims to explore ZER as a natural hepatic protective compound against ZEA-induced liver injury.

## 2. Materials and Methods

### 2.1. Drugs and Chemicals

The components, Zerumbone and Zeralenone, and a deoxynucleotidyl transferase dUTP nick end labeling (TUNEL) staining kit (Roche) were purchased from Sigma-Aldrich (St. Louis, MO, USA). The assay kits for Alkaline Phosphatase [17], Aspartate Aminotransferase (AST), Alanine Aminotransfer-ase (ALT), Catalase (CAT), Superoxide dismutase (SOD), Reduced glutathione (GSH), Lactate dehydrogenase [18], and Malondialdehyde (MDA) were procured from Nanjing Jiancheng Bioengineering Institute (Nanjing, China). The ELISA kits for analyzing IL-6, IL-1β, and TNF-α in mice tissues were purchased from Shanghai Haling Biological Technology Co., Ltd. (Shanghai, China). Only high purity reagents were used.

### 2.2. Animals

The study used 24 albino male mice (6 weeks old) of about 25–30 gm of weight. These were procured from Charles River Laboratories, Écully, France. The test subjects were maintained under regular laboratory atmospheric conditions, with a temperature of 22 ± 2 °C and a 12/12 h light/dark cycle. They were given access to ample food and water. The care and maintenance of investigational animals were in compliance with the guidelines, ethical policies and procedures approved by the ethics committee of the King Faisal University (Approval No: 71564).

### 2.3. Experimental Method

The subjects were selected randomly and divided into four groups, with six mice in each group. The first group was deemed the control group and was treated with DMSO (5% *v*/*v*). The second group was given ZEA (40 mg/kg wt) orally; this began from the second week and was followed till the end of the treatment process. ZER (15 mg/kg wt) alone was administered to the third group of animals for the first seven days; following this, each animal was treated with oral gavage (without anesthesia) with ZER (15 mg/kg wt) 6 h later and then ZER (15 mg/kg wt) for 4 weeks. The fourth group served as the drug control, and animals were treated with ZER 1st week to the end of the experiment.

When the experiment was completed, the mice were sedated with ether and culled. A cardiac puncture was made to collect their blood, following which the serum was separated and stored at −80 °C for analyzing pro-inflammatory cytokines and other biochemical parameters. A sensitive balance (Nimbus, MK, UK) was used to weigh the livers after excision. The biochemical parameters were assessed after homogenizing the livers in 0.1 M Tris–HCl buffer (pH 7.4).

### 2.4. Detection of Biochemistry Indexes

The liver tissues were processed to produce 10% homogenates by putting them in nine-fold (*w*/*v*) cold saline on ice and centrifuging them at 12,000 rpm for 30 min at 4 °C. Biochemical analysis of tissue enzymes. Tissue homogenates (10%) were centrifuged at 3000 rpm for 10 min at 4 °C to obtain the supernatant for the determination of lipid peroxidation, GSH, catalase (CAT), and superoxide dismutase (SOD). Lipid peroxidation levels were analyzed using a commercial kit based on the formation of thiobarbituric acid reactive substances in conjugation with malondialdehyde (MDA) following the method of Ayidin et al. [19]. The GSH levels were assayed using a commercial kit based on the formation of 5-thiol-2-nitrobenzoic acid. The activities of antioxidant enzymes (SOD, CAT, and GPx) were determined using commercially available assay kits following the instructions provided by the manufacturer (Pure One Biotechnology Ltd. Shanghai, China) according to the method of Bacanli et al. [20]. The biochemical results were spectrophotometrically analyzed using an UV-Vis digital spectrophotometer (Shimadzu UV-1900, Duisburg, Germany). The activity of lactate dehydrogenase [18] in serum was assayed using a known procedure as described. The activities of serum biomarker enzymes for liver damage such as ALP, AST, and ALT were analyzed using commercial kits according to the instructions given by the manufacturer, following the guidelines of Gnanaraj et al. [21] and Li et al. [22]. The results for serum biomarker enzymes were given as units per liter of serum. Total protein concentration in the liver tissues was measured by a standard Bradford protein assay.

### 2.5. ROS Assay

The liver tissues were analyzed for the ROS levels using a fluorescence probe, 2′,7′-dichlorodihydrofluorescein diacetate (H2DCFDA). To derive the supernatants for the assay, the tissues were homogenized in saline solution and centrifuged 10,000× *g* for 15 min at 4 °C. A 5-micromole probe, H2DCFDA dissolved in PBS, was added to the supernatants at 37 °C for 20 min and DCF fluorescence distribution was measured using Guava EasyCyte (Millipore, Burlington, MA, USA).

### 2.6. Western Blot

In all the samples, Bio-Rad protein assay helped ascertain the concentration of pro-tein and bovine serum albumin (BSA) in order to be used as the standard of reference. On the other hand, equal quantities (50 μg) of protein were resolved using SDS-PAGE (8–15%) before being moved across to nitrocellulose membranes. On the other hand, the mem-branes were blocked for half an hour at room temperature using 5% skimmed milk, prior to being incubated for a couple of hours using primary antibodies. The membranes were developed with an improved chemiluminescence substrate. Meanwhile the samples were examined using an LI-COR chemiluminescence imaging system(Lincoln, NE, USA) [2].

### 2.7. Histopathological Analysis

The liver tissues were prepared for histopathological analysis by fixing them in 10% formalin and embedded in paraffin. These lobes were sliced into 4-μm-thick sections and stained with H&E. The prepared sections were studied under an optical microscope. The ImageJ v1.8.0 software (National Institutes of Health, Bethesda, MD, USA) helped identify the areas of necrosis and inflammatory infiltration in the prepared tissues.

### 2.8. Measurement of Inflammatory

Cytokines IL-1β, IL-6 and TNF-α level in serum and tissue were determined by enzyme-linked immunosorbent assay [23] as per the manufacturer’s recommended procedure of the ELISA kits (R&D Systems, Minneapolis, MN, USA).

### 2.9. TUNEL Assay

The TUNEL assay is used to detect apoptosis by identifying DNA fragmentation in cells. The liver tissues in this study were stained with TdT-dUTP-fluorescein (Roche, Mannheim, Germany) [24] and studied under an optical microscope.

### 2.10. Statistical Analysis

Data are expressed as the mean ± SEM (Standard Error of Mean). A Student’s *t*-test calculated the statistical significance of the differences between the treatment groups and the control; * *p* < 0.05, # *p* < 0.05 was considered statistically significant. * *p* < 0.05 represents significant variations in the ZEA alone group compared with the control group. # *p* < 0.05 represents significant variations compared the ZEA alone and ZER with ZEA treatment groups.

## 3. Results

### 3.1. ZER Inhibits the Transaminases’ Activity

Alanine aminotransferase (ALT), aspartate aminotransferase (AST), and alkaline phosphatase [17] are the most commonly used biomarkers of liver damage. Among these, aspartate aminotransferase (AST) is the most sensitive predictor of liver pathology, as shown by recent studies. However, the results obtained by using AST/ALT ratio to predict cirrhosis can vary [13,25]. Therefore, we analyzed the mice serum to determine the alterations in enzyme activity. Figure 1C–E show that the serum activity of ALT, AST, and ALP was markedly elevated in the group challenged with ZEA (*p* < 0.05), indicating that ZEA infection was successfully established. Interestingly, pretreatment with ZER effectively brought down the serum activity of ALT, AST, and ALP. These results suggested that ZEA-induced liver disorders can be controlled with ZER owing to the latter’s hepatoprotective properties. Figure 1F,G and Table S1 show that the body and liver weight showed no significant variation between the control group and test subjects.

### 3.2. ZER Suppressed the ZEA-ROS Generation in Hepatic Tissues

ROS can modulate several inflammatory mediators [26]. It is interesting to note that ZEA has been found to induce ROS in various cellular models in vitro and subsequent oxidative DNA damage [27,28,29]. The DCFH2-DA fluorescence method was used to investigate intracellular ROS’ role in ZEA-induced liver injury. Moreover, data indicated that ZER pretreatment significantly attenuated the ZEA-induced ROS accumulation (Figure 1H).

### 3.3. ZER Inhibited ZEA-Induced Pro-Inflammatory Cytokine Secretion in Mice Serum

**Figure 2:**
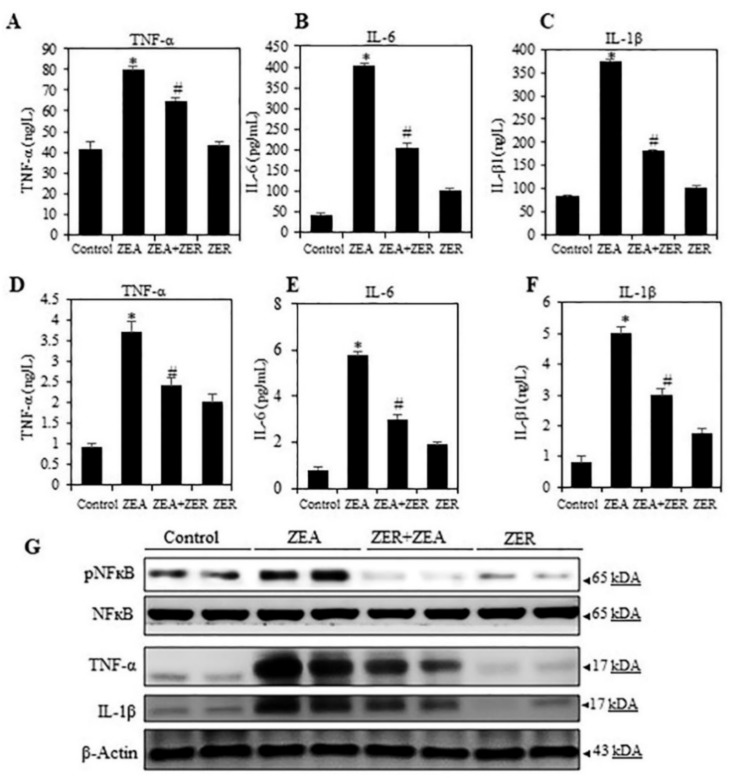
ZER curbing pro-inflammatory cytokine production by modulating the NF-κB. Effect of ZER on ZEA induced serum secretions of (**A**) TNF-α, (**B**) IL-6 (**C**) IL-1β were measured through commercial ELISA kits. Effect of ZER on liver tissues inflammatory responses (**D**) TNF-α, (**E**) IL-6 (**F**) IL-1β were measured through commercial ELISA kits (cytokines were measured in hepatic protein levels). Western blotting showing the changes in liver tissues of control and experimental groups: (**G**) NFκB (p65), TNF-α, and IL-1β levels. Data are expressed as the mean values ± SEM of independent experiments (*n* = 6). * *p* < 0.05 represents significant variations in the ZEA alone group compared with the control group. # *p* < 0.05 represents significant variations compared the ZEA alone and ZER with ZEA treatment groups.

The transcription factor NF-κB regulates several innate and adaptive immune functions. It is also an important inflammatory response mediator [30]. Thus, we analyzed if NFκB suppressed inflammation after ZER pretreatment in ZEA-infected mice serum and liver tissues. First, the mice serum and liver tissues were studied for the effect of ZER pretreatment on the pro-inflammatory cytokines such as TNF-α, IL-6, and IL-1β. Data obtained from the serum and liver showed a stark rise in the pro-inflammatory cytokines in mice challenged with ZEA (*p* < 0.05) (Figure 2A–F). Moreover, it was observed, as in the case of data on ROS, ZER pretreatment significantly attenuated the ZEA-induced cytokines.

Then the effect of ZER pretreatment on the expression patterns of NF-κB, TNF-α IL-6, and IL-1β was tested. Western blotting showed that ZEA stimulation overexpressed the NF-κB, TNF-α IL-6, and IL-1β (Figure 2G). However, ZER suppressed the transcriptional activation of p65, further suppressing inflammatory enzymes and cytokine expressions. This proves that ZER can inhibit pro-inflammatory cytokines induced by ZEA.

### 3.4. ZER Inhibit(s) the LACTATE Dehydrogenase Activation

**Figure 3:**
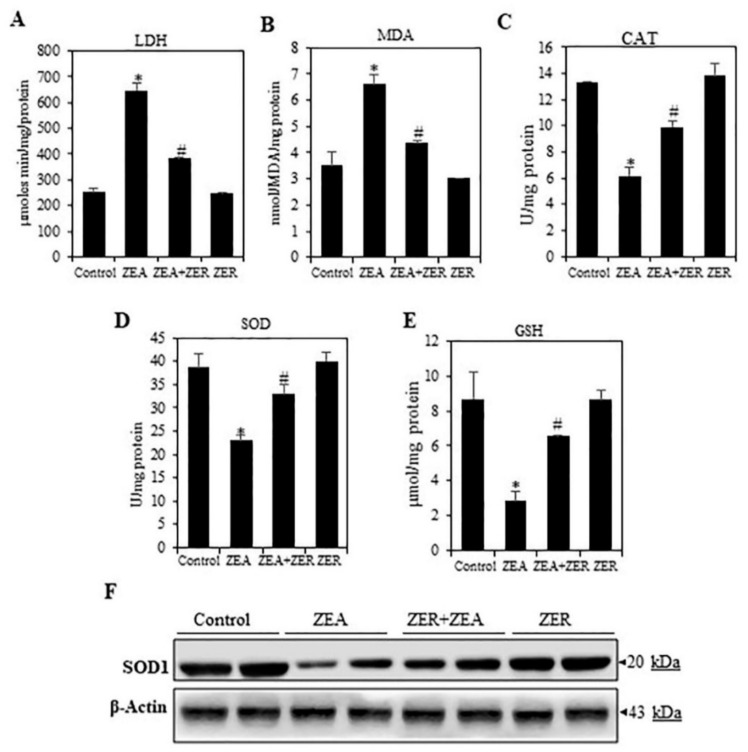
Effect of ZER on ZEA-induced MDA formation and antioxidant in mouse liver tissue. (I: control; II: ZEA alone; III: ZER with ZEA; IV: ZER alone). (**A**) LDH (Serum) (μ/min/mg protein), (**B**) MDA [26] (nmol/MDA/mg protein), (**C**) SOD (U/mg protein), (**D**) CAT (U/mg protein), and (**E**) GSH (µmol/mg protein). ELISA kits were used in compliance with the manufacturer’s directions. (**F**) Western blotting analysis of SOD1 expression in the liver of control and experimental groups. Data are expressed as the mean values ± SEM of independent experiments (*n* = 6). * *p* < 0.05 represents significant variations in the ZEA alone group compared with the control group. # *p* < 0.05 represents significant variations compared the ZEA alone and ZER with ZEA treatment groups.

Ferriero and colleagues have recently shown that hepatic injury is associated with nuclear translocation of LDH and pyruvate dehydrogenase complex, resulting in lactate production from pyruvate in the nucleus, as well as increased nuclear acetyl-CoA; in turn, hyper-acetylation of histone H3 takes place, upregulating the genes associated with the damage response, leading to cell death [31]. We found a significant rise in the LDH level in mice treated with ZEA only (Figure 3A). The ZER-pretreated group showed a marked decrease in LDH levels as compared with the ZEA group. The results clearly show that ZER pretreatment can inhibit ZEA-induced hepatotoxicity.

### 3.5. ZER Activates Antioxidant Enzymes

A crucial mechanisms by which ZEA induces liver injury is by causing oxidative stress [2,32]. Increased ROS production in hepatocytes caused by ZEA depletes GSH and peroxidizes lipids. Lipid peroxidation forms MDA, a biomarker of oxidative stress.

ROS cause damage by decreasing the antioxidant activity of enzymes such as SOD and CAT. To analyze the oxidative stress parameters, the MDA and GSH levels and SOD and CAT activity were analyzed. The GSH and SOD and CAT activity were remarkably increased and MDA levels decreased in the ZEA-challenged group (Figure 3B–D); moreover, these parameters attained a normal level in the ZER-pretreated group. Further, Western blotting revealed that ZER induced the activation of SOD1 in ZEA-challenged liver tissue (Figure 3F). Thus, ZER exhibits hepatoprotective properties is by alleviating oxidative stress.

### 3.6. ZER Activation of Cell Survival Proteins

**Figure 4:**
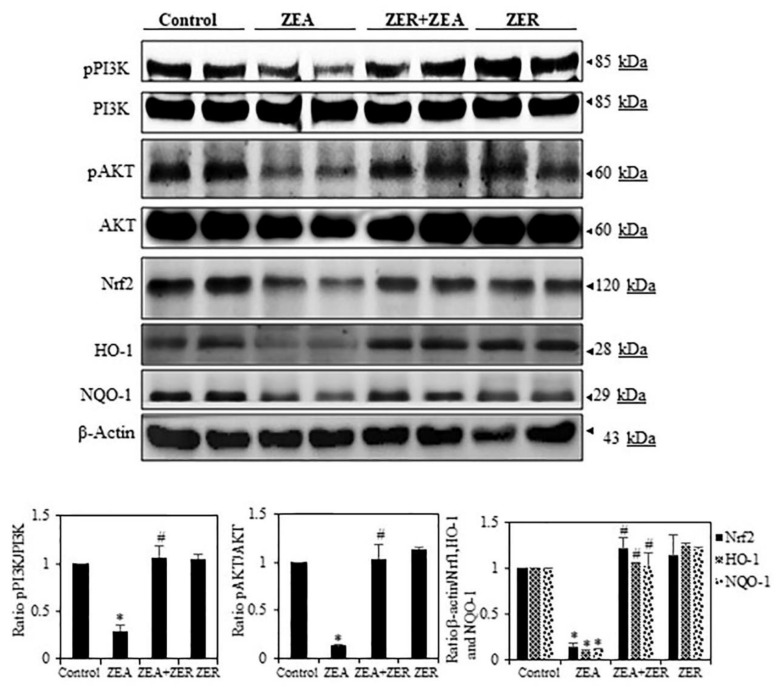
Effect of ZER on PI3K/AKT phosphorylation and Nrf2 activation in mouse liver tissue. Expression of pPI3K, pAKT, Nrf2, HO-1, and NQO-1 proteins in albino mice. Total PI3K and Akt and β-Actin levels were measured as loading controls. Data are expressed as the mean values ± SEM of independent experiments (*n* = 6) * *p* < 0.05 represents significant variations in the ZEA alone group compared with the control group. # *p* < 0.05 represents significant variations compared the ZEA alone and ZER with ZEA treatment groups.

PI3K/AKT activation induces different cell survival mechanisms. Moreover, through phosphorylation, PI3K/AKT activates the transcription factor cyclic AMP response element-binding protein (CREB) and the IκB kinase (IKK), a positive regulator of NF-κB, both of which regulate gene expression and anti-apoptotic activity [33]. To this end, we also analyzed whether ZER could induce phosphorylation of ZEA-inhibited PI3K/AKT in hepatic tissues. Figure 4 depicts how ZEA-challenged liver tissue showed inhibition of phosphorylation of PI3K/AKT, whereas pretreatment with ZER increased phosphorylation of this protein. These results clearly indicate that ZER confers a significant protective effect against ZEA-induced liver damages.

### 3.7. ZER Upregulates HO-1 and NQO-1 Expression via Nrf2 Activation

The activation of the cis-acting antioxidant response element (ARE) is dependent on the transcription factor Nrf2. Nrf2 activates several antioxidant genes that eliminate oxidative stress in cellular systems, such as hemeoxygenase-1 (HO-1), NAD(P)H-quinone oxidoreductase-1 (NQO-1), and GSH. Since these enzymes act by exhibiting cytoprotective, antioxidant, and anti-inflammatory properties [34], we had initially hypothesized that ZER arrests the ZEA-induced oxidative stress and apoptosis by inducing antioxidant genes such as HO-1 and NQO-1 and its transcription factor Nrf2. Our study results confirmed our hypothesis by showing that ZER significantly increased HO-1, NQO-1, and Nrf2 expression (Figure 4).

### 3.8. ZER on Histopathological Effects

**Figure 5:**
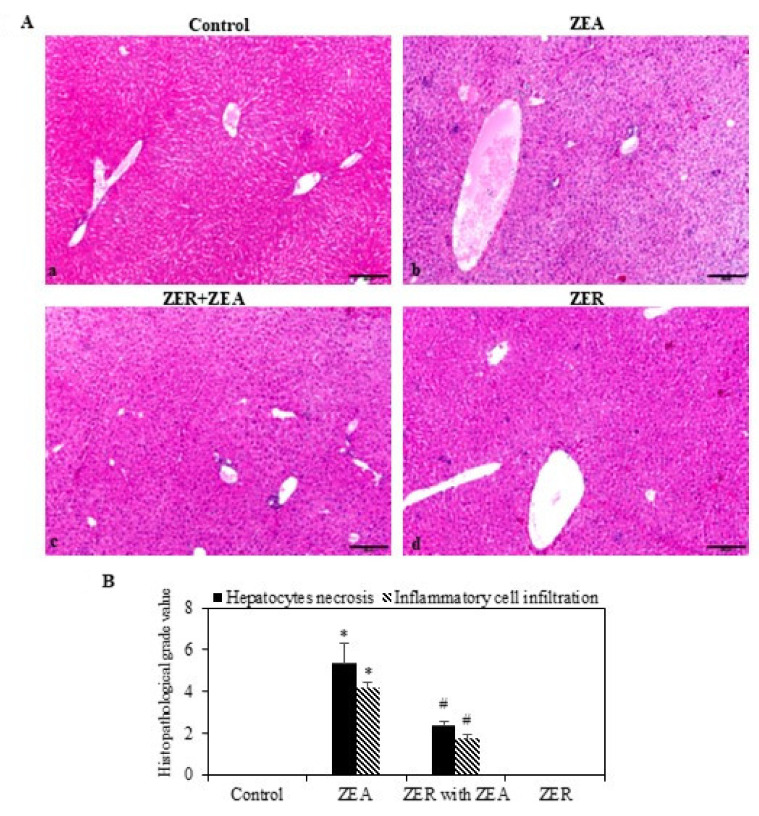
Effect of ZER on ZEA-induced liver histopathological changes. H&E-stained mouse liver tissue section after treatment with ZEA alone or with both ZER and ZEA: (**Aa**) control, hepatocyte cords (Hc), clearly visible nuclei of hepatocytes; (**Ab**) ZEA-challenged showing infiltrated inflammatory cells; (**Ac**) ZER with ZEA showing lesser sinusoidal congestion and some infiltrated inflammatory cells; and (**Ad**) ZER treatment alone showing normal hepatocytes. (**B**) Histopathological representation of hepatocyte necrosis and inflammation; value *n* = 6. * *p* < 0.05 represents significant variations in the ZEA alone group compared with the control group. # *p* < 0.05 represents significant variations compared the ZEA alone and ZER with ZEA treatment groups.

The liver tissues were stained with H&E for identification of histopathological alterations caused by ZEA (Figure 5). The central lobular areas, about 49% of the tissue section, evidenced moderate hepatocytic necrosis (Figure 5A in panel b). Nevertheless, the necrosis slowed down when ZER was administered (Figure 5B). Thus, the histopathologic examination confirmed that ZER pretreatment effectively alleviated ZEA-induced centrilobular necrosis.

### 3.9. Fragmentation of Apoptotic DNA Inhibited by ZER in ZEA Induced Mouse Model

**Figure 6:**
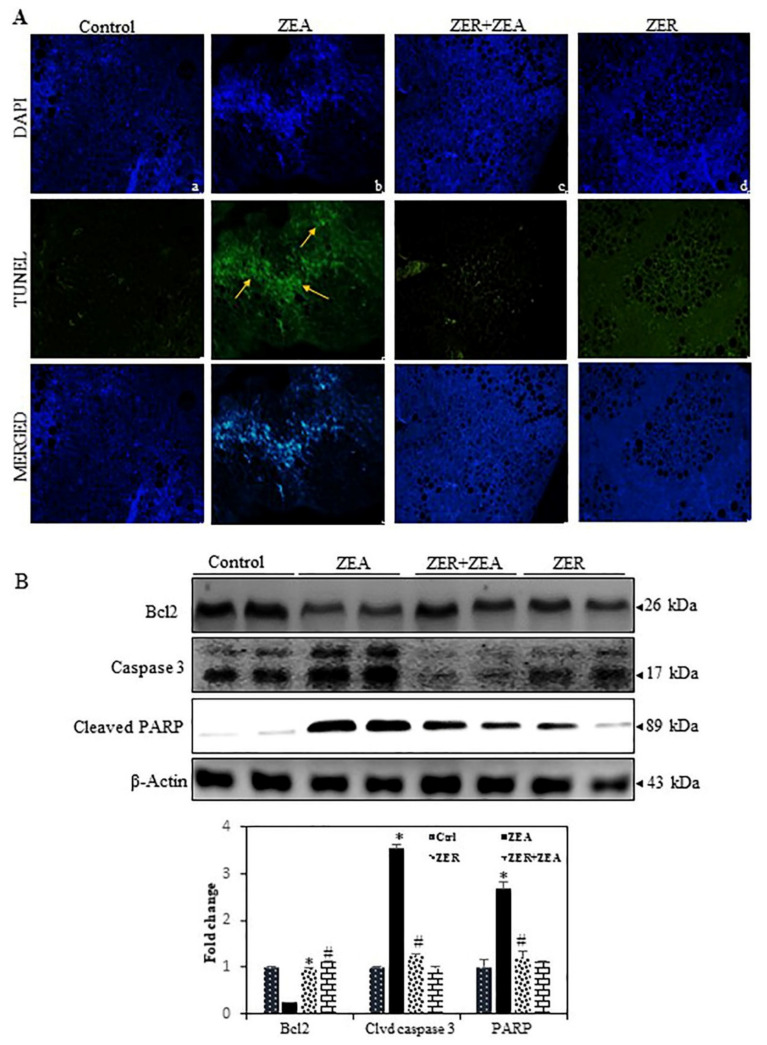
Anti-apoptotic effect of ZER on ZEA-induced liver tissue of control and test groups. (**A**) TUNEL assay for untreated cellophane exhibiting apoptotic cells in liver tissue sections: (**a**) control, (**b**) ZEA-challenged, (**c**) ZEA treated with ZER, (**d**) ZER alone. (**B**) Western blotting showing expression of Bcl-2, caspase-3, and cleaved PARP (clvd PARP) protein. * *p* < 0.05 represents significant variations in the ZEA alone group compared with the control group. # *p* < 0.05 represents significant variations compared the ZEA alone and ZER with ZEA treatment groups.

The higher number of TUNEL-positive cells in ZEA-challenged mice than the control group suggested that ZEA arrested cell growth but increased apoptosis (Figure 6A). The assay also showed how pretreatment with ZER significantly reduced TUNEL-positive cell count.

### 3.10. ZER Downregulates the Dysregulates Caspase-3 and Activation of the Bcl-2 Expression Induced by ZEA

Caspase-3 is a major effector and influences characteristic apoptotic events, including chromatin condensation, DNA fragmentation, and apoptotic body formation [35]. Therefore, we hypothesized that the ZEA-induced DNA damage and apoptosis are mediated by the activation of caspase-3 and cleaved PARP. As shown in Figure 6B, ZEA remarkably increased caspase-3 activation and cleaved PARP in the hepatic tissue, whereas ZER significantly inhibited caspase-3 activation and cleaved PARP. Bax, a proapoptotic protein, is upregulated by mycotoxin and that Bcl-2, an anti-apoptotic protein, is suppressed [36]. In line with this, we observed that ZEA exposure markedly decreased Bcl2 expression (Figure 6B). In contrast, ZER pretreatment was found to upregulate Bcl-2 in hepatic tissues. These data strongly suggest that hepatoprotection of ZER against ZEA-induced apoptosis is due to the activation of caspase-3 and dysregulation of Bcl-2.

## 4. Discussion

ZEA is known to cause liver damage in animals, as well as human beings. This study aimed to determine whether ZER could alleviate ZEA-induced changes via ameliorating inflammation and oxidative stress-induced apoptosis. Our findings showed that ZEA markedly increased the levels of serum hepatic enzymes such as AST, ALT, and ALP and LDH. These effects may be attributed to the hepatorenal injury induced by ZEA. Transaminases (AST and ALT) are biomarkers of liver damage, and ALT is considered a gold indicator of liver injury [37]. The increased levels of these enzymes may be due to dysregulated permeability of the liver membrane and/or disturbed biosynthesis of these enzymes, leading to increased levels of circulating enzymes [38]. Additionally, ALP activities may increase due to obstruction of the bile duct as a consequence of the swelling and hypertrophy of hepatocytes induced by ZEA [39]. Elevations of these enzymes in the serum may cause pathologic changes in the liver, which may decrease the biosynthesis of total protein and albumin.

Our previous study reported that ZER is known to have significant antioxidant properties [9]. ZER decreases the AST and ALT activity in serum and arrests GSH depletion induced by hepatotoxins of CCl4 in the liver of rats [16]. Therefore, we conclude that ZER can prevent hepatic damage induced by oxidative stress. Recently, researchers have demonstrated the effects of individual and mixtures of Fusarium toxins on the release of pro-inflammatory cytokines and the modulation of secretory mucins, as well as total mucin-like glycoprotein secretion, all of which are essential components of host mucosal immunity [40]. Our data also showed that ZEA leads to an enhanced expression of cytokines such as TNF-α, IL-6, and IL-1β in mouse serum and liver tissues. It was also reported that ZEA increased the expression of pro-inflammatory pro-TNF-α, IL-1β in pig spleens [41]. Our histopathological analysis also showed how ZER pretreatment curbed ZEA-induced intestinal inflammatory response by suppressing inflammatory cytokines. Similarly, Western blotting indicated that ZEA stimulated the expression of nuclear p65, iNOS, COX-2, TNF- α, and IL-1β proteins; however, ZER pretreatment attenuated this effect. It was found that ZER blocked the transcriptional activation of p65, thereby suppressing inflammatory enzyme and cytokine protein expression.

Whenever a tissue injury occurs due to oxidate stress, the body releases antioxidant enzymes such as SOD, CAT, and GSH, which scavenge ROS in the liver [42]. Studies have shown that ZER prevents tissue injury by curbing the production of ROS [43,44,45]. The same mechanism is followed when paracetamol’s cytotoxicity results in liver injury due to ROS generation [46]. However, ZER can be a contraindication in certain special cases such as colorectal cancer and melanoma, where ZER has been found to remarkably increase the ROS levels in the cells [47,48]. This contradictory finding could possibly be due to the variation in cell function in the selected cases; for instance, ZER favoring ROS generation could possibly be due to its effect on apoptosis in cancer [49], whereas ZER curbing ROS generation can be found in inflammatory diseases such as hepatic disease. Under oxidative stress, the PI3K/Akt signaling pathway leads to Nrf2-dependent transcription and overexpression of HO-1 protein [50,51]. We found that phosphorylation of AKT protein increased in the presence of ZER, which possibly upregulated ZER-induced Nrf2/HO-1 protein. Under normal physiological conditions, Keap1 sequesters Nrf2 in the cytoplasm. After electrophilic agents or ROS oxidize cysteine residues within Keap1, Nrf2 translocates to the nucleus following dissociation from its cytoplasmic docking protein. In the nucleus, Nrf2 activates the transcription of several phase II detoxifying enzymes and antioxidant enzymes including HO-1 by binding to their promoter regions [51,52]. However, it is not known whether PI3K/AKT signaling pathway is also involved in ZER-stimulated Nfr2 activation in hepatic tissues exposed to ZEA. We found that AKT phosphorylation was significantly increased in ZEA-challenged liver tissues that received ZER treatment. Further, a proportional correlation was observed between PI3K/Akt signaling and the antioxidant effect and hepatoprotective property of ZER—inhibition of the former led to arresting of the latter. Another finding was that intracellular ROS and MDA production due to ZEA was curbed by ZER. We further investigated the signaling mechanisms responsible for the activation of HO-1, as it was believed that the latter influences ZER’s hepatoprotection significantly. It is believed that HO-1 is a target gene of Nrf2, a redox-sensing transcription factor that responds to oxidative stress by controlling the expression of various antioxidant enzymes. Reports have shown that HO-1 is significantly reduced in Nrf2-deficient mice, and that Nrf2 disruption stops HO-1 gene induction [53]. HO-1 converts heme to biliverdin, free iron, and carbon monoxide. HO-1 works through upregulation to enhance cellular resistance to stress. Furthermore, HO-1 protects HepG2 cells from H2O2 and hypoxia-induced hepatotoxicity [54]. This study found that ZER treatment increased the levels of HO-1 via Nrf2, thereby increasing the activity of antioxidant enzymes such as SOD, CAT, and GSH, which inhibits or prevents ZEA-induced ROS generation.

Hepatic apoptosis and fibrosis are salient causative factors of hepatic remodeling and liver failure [55]. In this study, the TUNEL staining and Western blot analysis showed that ZER suppressed apoptosis in ZEA-challenged mice through attenuation of key apoptotic mediators, cleaved-caspase3 and PARP. Furthermore, ZER enhanced the expression of the survival markers such as Bcl-2. Thus, it can be concluded that ZER prevents hepatic injury through attenuation of apoptosis and enhancement of the survival pathway. These findings are in line with our previous reports demonstrating how kaempferol suppressed the apoptotic protein Caspase-3 and enhanced the anti-apoptotic protein Bcl-2 to attenuate hepatic apoptosis [2].

## 5. Conclusions

In summary, this study’s results suggest that ZER protects hepatic tissue against ZEA-induced oxidative stress cell damage. ZER inhibited ROS and NFκB expressions, thereby leading to the suppression of pro-inflammatory TNF-α, IL-6, and IL-1β secretions in the ZEA-stimulated mice serum and liver tissues. ZER’s anti-inflammatory and antioxidant properties induce antioxidant genes HO-1 and NQO-1 via the Nrf2 signaling pathways. The ZEA-induced intracellular oxidative stress and apoptosis were inhibited by ZER through activation of the PI3K/AKT-mediated Nrf2/HO-1 signaling pathway in albino mice liver tissue. Therefore, ZER can be used as a therapeutic drug to prevent hepatic damage. Further studies must study the possible therapeutic application of ZER to treat liver toxicity in humans.

## Data Availability

Data is contained within the article.

## Supplementary Materials

The following are available online at https://www.mdpi.com/article/10.3390/antiox10101593/s1. Table S1: Body weight tracker through throughout experimental period.
