# Supplementary material for: Zerumbone Exhibit Protective Effect against Zearalenone Induced Toxicity via Ameliorating Inflammation and Oxidative Stress Induced Apoptosis
Source: Antioxidants (Basel). 2021 Oct 12;10(10):1593. doi: 10.3390/antiox10101593 (PMC8533127; doi:10.3390/antiox10101593)
Supplement: Supplementary file 1 [file antioxidants-10-01593-s001.zip › antioxidants-1397190-supplementary.pdf]

## Supplementary Table S1

**Table S1.** Body weight tracker through throughout experimental period.

| | Control | ZEA | ZER +ZEA | ZER |
|---|---|---|---|---|
| **Week 1** | 24.11±0.33 | 23.92±0.81 | 24.20±0.10 | 24.52±0.08 |
| **Week 2** | 22.36±0.12 | 24.71±0.10 | 24.866±.086 | 24.506±0.05 |
| **Week 3** | 23.53±0.08 | 25.53±0.14 | 26.266±0.13 | 25.806±1.30 |
| **Week 4** | 25.45±0.12 | 23.30±0.14 | 23.42±0.14 | 25.622±0.24 |
| **Week 5** | 24.70±0.10 | 25.16±0.01 | 22.404±0.04 | 25.456±0.05 |
| **Week 6** | 24.57±0.08 | 24.26±0.11 | 22.804±0.08 | 25.19±0.12 |

Mice were sampled 6 weeks after start of treatment; Values represent means +SD; Unit gm)
